# Supplementary material for: Prevalence of soil-transmitted helminths infection and associated risk factors among residents of Jigjiga town, Somali region, Eastern Ethiopia
Source: PLoS One. 2025 Jan 30;20(1):e0317829. doi: 10.1371/journal.pone.0317829 (PMC11781731; doi:10.1371/journal.pone.0317829)
Supplement: Attachment — Submitted filename: Comments to author(s) 2.docx [file pone.0317829.s002.docx]

I'm pleased to have the opportunity to review this manuscript. Now, I finished reviewing the manuscript titled "Prevalence of Soil-Transmitted Helminths Infection and Associated Risk Factors among Residents of Jigjiga Town, Somali Region, Eastern Ethiopia." I found the title of the study to be interesting. However, I recommend revisions before it can be considered for publication. I have provided major comments on the methodology, results, and discussion sections that need revision. Additionally, some minor comments regarding the abstract, introduction, and declaration need modification.

**General comments**

- The paper lacks page number.
- Parasite names are not written in scientific method (italic) throughout the document.
- Citation does not align with journal guidelines.

**Abstract**

**Method**: Mention sampling method

**Results**: “STH prevalence 33 was 11.4% overall (95% CI = 9.0, 14.0)” vague sentence paraphrase it.

**Conclusion**: You wrote the result again; please add a recommendation based on your finding.

**Introduction**

- Your citation does not align with the guidelines of the journal. Remove the superscript and write as follows: “[1]”

**Methodology**

**Study area:** add health facility information of the Jigjiga Town

**Sample technique/Procedures**

- The sampling technique is the heart of one study; missing this crucial methodology section indicates that the study wasn’t conducted properly. Also, the readers of the article understand the study clearly. I strongly recommend the author add this section and procedure support with a chart or figure unless this article is rejected.

1. Total kebeles in Jiggiga town
2. Name six selected kebeles of the town by which method.
3. The total eligible population (household) in selected kebeles
4. Source of study participants (household)
5. Proportional allocation (number of participants or household selected from each kebele)
6. Indicate the sampling method applied
7. If systematic, indicate K-value.
8. Mention whether directly selected the participants or their household.
9. As I understood from your study conducted on households of selected kebele, if there were more than one eligible participant in the household, how did you select them?

- Whoever gathers data from the participant, please mention them.
- Did the author use the English-language version of the questionnaire for data collection? Please indicate it.
- The author mentioned “a pre-test was made on 28 individuals (5%) in one Jigjiga town kebele other than the sampled study study kebele." Name that kebele.

**Results**

- Remove this paragraph from the result section and incorporate it into the methodology ology section. “As recommended by WHO, egg counts were used to classify the intensity of infection as light, moderate, and heavy infection, respectively for A. lumbricoides 1-4999epg, 5000-49999epg, and ≥50000epg; for hookworms 1-1999epg, 2000-3999, and ≥4000; for T. trichiura 1-999epg, 1000-9999epg, and ≥10000epg (91).”
- The interpretations of AOR are inconsistent, e.g., three times (AOR = 3.19), and 2.3 times (AOR = 2.32). The author should familiarize them and also revise by adding nearly or other appropriate terms.

**Discussion**

- This study finding is greater than your study result, “Thailand's Chachoengsao province 14.3%.” Check it.
- I recommend the author not repeatedly write results in discussion. AOR results, e.g., (AOR = 2.32; 95% CI = 314 1.12, 4.79; p = 0.023), should be removed from discussion.

**Declaration**

**Author contributions**

- This sentence was incomplete. “Every author has significantly contributed to the concept, method of study, data collection, analysis, and interpretation of the”
- Similarly, this sentence is also so incomplete. “They also contributed to the manuscript's development, critically reviewed, and decided which publication the paper should be submitted to.”
